# The Cuba–United States Thaw: Building Bridges through Science and Global Health

**DOI:** 10.4269/ajtmh.17-0136

**Published:** 2017-06-07

**Authors:** Daniel G. Bausch, Vivian Kouri, Sonia Resik, Belsy Acosta, Gerardo Guillen, Karen Goraleski, Marcos Espinal, Maria G. Guzman

**Affiliations:** 1 American Society of Tropical Medicine and Hygiene, Oakbrook Terrace, Illinois; 2 Tulane School of Public Health and Tropical Medicine, New Orleans, Los Angeles; 3 Instituto de Medicina Tropical Pedro Kourí, Havana, Cuba; 4 Centro de Ingenieria Genetica y Biotecnologia, Havana, Cuba; 5 Pan American Health Organization, Washington, District of Columbia

## Abstract

Beginning in 2014, there has been significant progress in normalization of relations between Cuba and the United States. Herein, we discuss the history and recent progress in scientific collaboration between the two countries as well as the continued challenges. Science and global health diplomacy can be key tools in reestablishing a trusting and productive relationship of mutual and global benefit, bringing about better and healthier lives for people in both Cuba and the United States.

On December 17, 2014, U.S. President Barack Obama and Cuban President Raúl Castro announced the beginning of a process of normalizing relations, now commonly referred to as the “Cuban Thaw.”

On July 20, 2015, the two countries restored diplomatic relations, which had been severed in 1961 during the Cold War. This was followed in March 2016 by Obama's historic visit to the island—the first official visit of a U.S. president since the 1959 Cuban revolution. In December and also during his visit, Obama highlighted health as one of the shared priorities of the two governments. These events renewed optimism among many observers that the two nations, long estranged but only 90 miles apart, can continue to work together on many fronts, but especially in the domains of science and global health, which in and of themselves can serve as effective tools to normalize the relationship.

The American Society of Tropical Medicine and Hygiene (ASTMH) has contributed to this renewed engagement by bringing Cuban and American scientists together at the Society's 2015^1^ and 2016^2^ annual meetings to present on the many scientific advances coming from Cuba, continued challenges, and what the future can bring if the two countries can rekindle their history of cooperation in science and global health. A symposium at the 2015 meeting in Philadelphia was cosponsored by the Pan American Health Organization (PAHO) with participation from members of the U.S. National Institutes of Health (NIH) and U.S. Department of Health and Human Services (HHS). Immediately after the ASTMH Meeting, a dinner was held in Washington, DC, hosted by the Center for Strategic and International Studies, at which representatives from the U.S. State Department, HHS, Uniformed Services University of the Health Sciences, PAHO, NIH, and ASTMH welcomed the Cuban visitors and eagerly discussed the potential of new collaborations. The next day the delegation visited the NIH in Bethesda, MD, the first visit by Cuban scientists to NIH in more than 50 years. ASTMH members also participated in a virology workshop organized by the Cuban Society of Microbiology and Parasitology in Havana in December 2016^3^ and the Society will cosponsor a scientific conference commemorating the 80th anniversary of Cuba's premier institute for tropical medicine, the Instituto Pedro Kourí, in Havana in December 2017.

Bilateral cooperation in science and global health between Cuba and the United States makes sense. Our geographical proximity means that we share waters and are affected by the same environmental factors, the same tropical storms, and many of the same health challenges. The United States and Cuba share an obvious interest in detecting and responding to emerging infectious diseases such as dengue, chikungunya, and Zika fevers, which are growing rapidly in the region and for which no vaccines currently exist. As travel and business opportunities grow between the two countries, so will the risk of transfer of pathogens. Mitigating that risk will require intensified collaboration and investment of resources. In addition, both countries have aging populations, necessitating an increased focus on biomedical research and health services for cardiac diseases, cancer, diabetes, and neurodegenerative diseases such as Parkinson's and Alzheimer's diseases.

Beyond their physical proximity, Cuba and the United States share a dedication to scientific inquiry and discovery. Despite having different health systems and priorities, both countries are proud of their scientific accomplishments and advances in public health. Cuba reports one of the lowest infant mortality rates in the world and a life expectancy on par with the United States. In 2014, Cuba became the first country to eliminate mother-to-child transmission of human immunodeficiency virus and syphilis. Cuba is one of the few countries without a shortage of human resources for health. They have ample numbers of trained medical personnel and have offered clinical support to respond to emergencies and assist with the implementation of health programs in more than 100 countries. Heberprot-P, a Cuban medication licensed in more than 20 countries, reduces risk for limb amputation among patients with diabetes by 70%. Early studies on CIMAVax, an experimental immunotherapeutic agent for lung cancer developed by the Cuban Center for Molecular Immunology in Havana, have indicated a survival benefit as well as improved quality of life. Products such as these could provide enormous health benefit to the U.S. population but have not generally been available for use in the United States. For example, in any given year, nearly 80,000 patients with diabetes in the United States undergo amputation at a cost of approximately $50 billion.^4^ Heberprot-P could potentially be of enormous benefit to these patients.

U.S. accomplishments in health and the biomedical sciences are widely acclaimed. The NIH is the world's largest funder of medical research and has the largest hospital dedicated solely to clinical research. NIH-funded research led to the development of magnetic resonance imaging technology, an understanding of how viruses can cause cancer, insights into cholesterol control, and knowledge of how our brain processes visual information, among many other advances. The U.S. Centers for Diseases Control and Prevention has established the Global Disease Detection Program with regional centers throughout the world that have responded to outbreaks of anthrax in Scotland, botulism in Thailand, cholera in Haiti, Ebola in west Africa, and dozens more.

The strengths and accomplishments of Cuba and the United States in the health sciences complement each other. Not surprisingly, some of the two countries' greatest health achievements have resulted from working together. At the turn of the 20th century, Cuban and American scientists such as Carlos Finlay and Walter Reed collaborated to unravel the mystery of yellow fever, confirming Finlay's theory of mosquito-borne transmission and promoting evidence-based control modalities. Over the years, determined Cuban and U.S. government scientists have found ways to engage, albeit limited and through a heavy layer of regulatory red tape, on a number of scientific research collaborations, including in the fields of health (such as infectious diseases, antimicrobial drug resistance, neuroscience, cancer, and migrant health), marine science, ecology and conservation, and atmospheric and geoscience research. In 2014, a Cuban brigade of more than 400 doctors and nurses staffed an Ebola Treatment Unit funded by the U.S. Agency for International Development in Monrovia, Liberia, helping to control the outbreak of Ebola virus diseases in West Africa. Cuban and U.S. physicians have also recently worked together on emergency responses in the aftermath of Hurricane Matthew in Haiti, with Cuban physicians working on the USNS Comfort, a U.S. Navy hospital ship that sailed to Port-au-Prince to provide free medical care.

Cracks in the long-standing barriers to scientific collaboration between Cuba and the United States are starting to occur. In June 2015, the HHS sent three participants to Cuba on the first government-led delegation on health and science. In October 2016, former HHS Secretary Sylvia Burwell traveled to Cuba, where she signed a Memorandum of Understanding on cancer control and explored additional opportunities for collaboration. Also in October 2016, the U.S. Departments of the Treasury and of Commerce announced amendments to the Cuban asset control regulations. These changes will facilitate the movement of Cuban-origin pharmaceutical applications through the U.S. Food and Drug Administration (FDA) review process and allow FDA-approved Cuban-origin pharmaceuticals to be imported, marketed, and sold in the United States. In 2016, the FDA approved the first clinical trial of a Cuban drug in the United States—a Phase I/II clinical trial of CIMAVax. For its part, Cuba may benefit from broader engagement with U.S. biomedical and research communities who may, for example, provide the latest data and biotechnological advances for optimal clinical management of diseases Cuban clinicians may encounter as part of the many Cuban medical brigades overseas. A meeting between NIH and Cuban scientists to coordinate the public health response to and research on Zika virus disease was held in Havana in November 2016. Nongovernmental entities, including academic institutions and professional societies such as ASTMH, have stepped in to play a unique role in improving Cuba–U.S. relations, including facilitation of educational exchanges for students and faculty.

The recent steps toward normalization of relations are promising, but many questions and challenges remain. Nothing between the two countries is easily separable from politics. Trust needs to be built, not taken for granted. Recent political changes in the United States bring new questions over the thaw's future. Will President Trump continue the easing of trade and travel started by Obama? Will Cuba's policy toward the United States alter with a new generation of leaders in coming years? Leadership in Cuba will inevitably give way to a new generation. As that change takes place, the United States must address whether to continue the trade embargo, which has been in place since 1960. Despite legislation signed by President Clinton in 2000 creating limited exceptions for medical supplies and devices, the embargo remains a major impediment to normalization of relations and more advanced scientific development for both countries.

While elected leadership may remain split on the way forward, public sentiment is softening. In recent polls, 62% of Americans said they favored normalizing relations with Cuba and lifting the embargo.^5^ Lifting the embargo was favored by 63% of Cuban-Americans in Miami-Dade County, FL, the area with the highest concentration of Cuban Americans in the United States.^6^ The sentiment for normalization of relations in Cuba itself is hard to gauge. Obama's actions in that regard over the past few years appear to have been generally welcomed by the populace, albeit with concerns over how Cuba can integrate into and benefit from a more global economic engagement without sacrificing many of its fundamental principles and public health achievements.

Advancing political understanding between Cuba and the United States will require high-level political will and leadership, cross-border partnerships, and sustained advocacy. Science and global health diplomacy are key tools in reestablishing a trusting and productive relationship of mutual and global benefit. Health has a universal appeal, with the potential not only to transcend politics, but to promote and advance constructive political discourse. The exchange of data and views at recent scientific conferences in Cuba and United States was a great place to start. As Cuban and American scientists, clinicians, and experts in global health, we must continue our dialogue and advocacy to keep open our communications and collaborations and to keep moving forward. We recognize that we have much to teach, learn, and gain from each other. It is our fervent hope that the political discourse continues to intensify in a positive direction to unlock the potential of Cuban and American science to bring about better and healthier lives for people in both countries.
